# Supplementary material for: Sleep decreases neuronal activity control of microglial dynamics in mice
Source: Nat Commun. 2022 Oct 21;13:6273. doi: 10.1038/s41467-022-34035-9 (PMC9586953; doi:10.1038/s41467-022-34035-9)
Supplement: Supplementary file 1 — Supplementary Information [file 41467_2022_34035_MOESM1_ESM.pdf]

Supplementary Figure 1

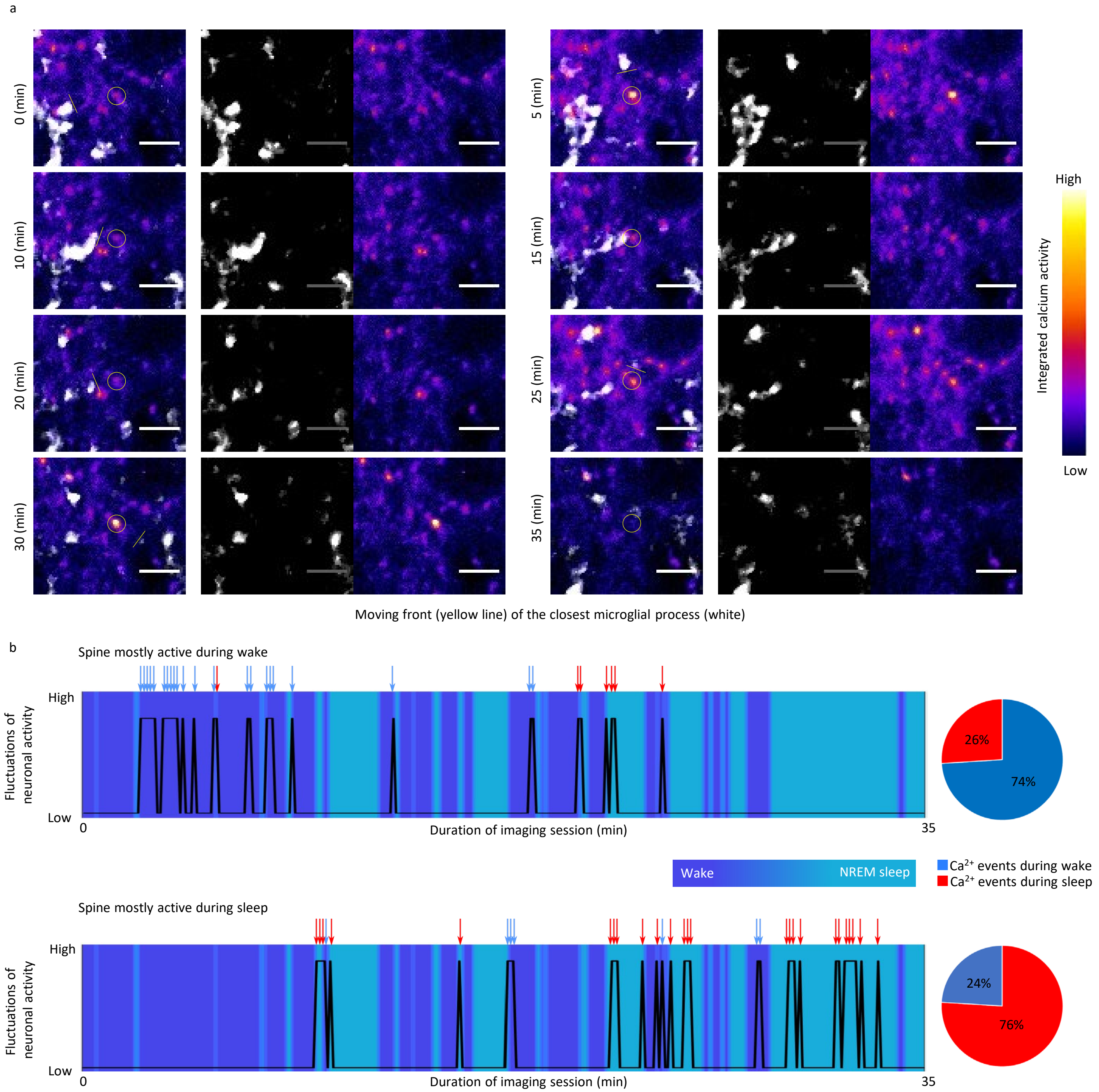

Supplementary Figure 1. Quantification of microglia-spine distance and classification of spines active during wake and sleep. (a) Analysis of microglial (gray scale) motility at the level of the spine (fire pseudo-color) by measuring the distance between the moving front of the closest microglial process (yellow line) and the spine (encircled). Images of each channel at each time point are shown alongside the color-coded image. Scale bar=10μm. Representative images from the analysis present in Figure 2b, replicated 68 times. **(b)** An example of a spine active during wake (upper panel) and a spine active during sleep (lower panel). The blue and red arrows indicate calcium events during wake and sleep, respectively.

Supplementary Figure 2

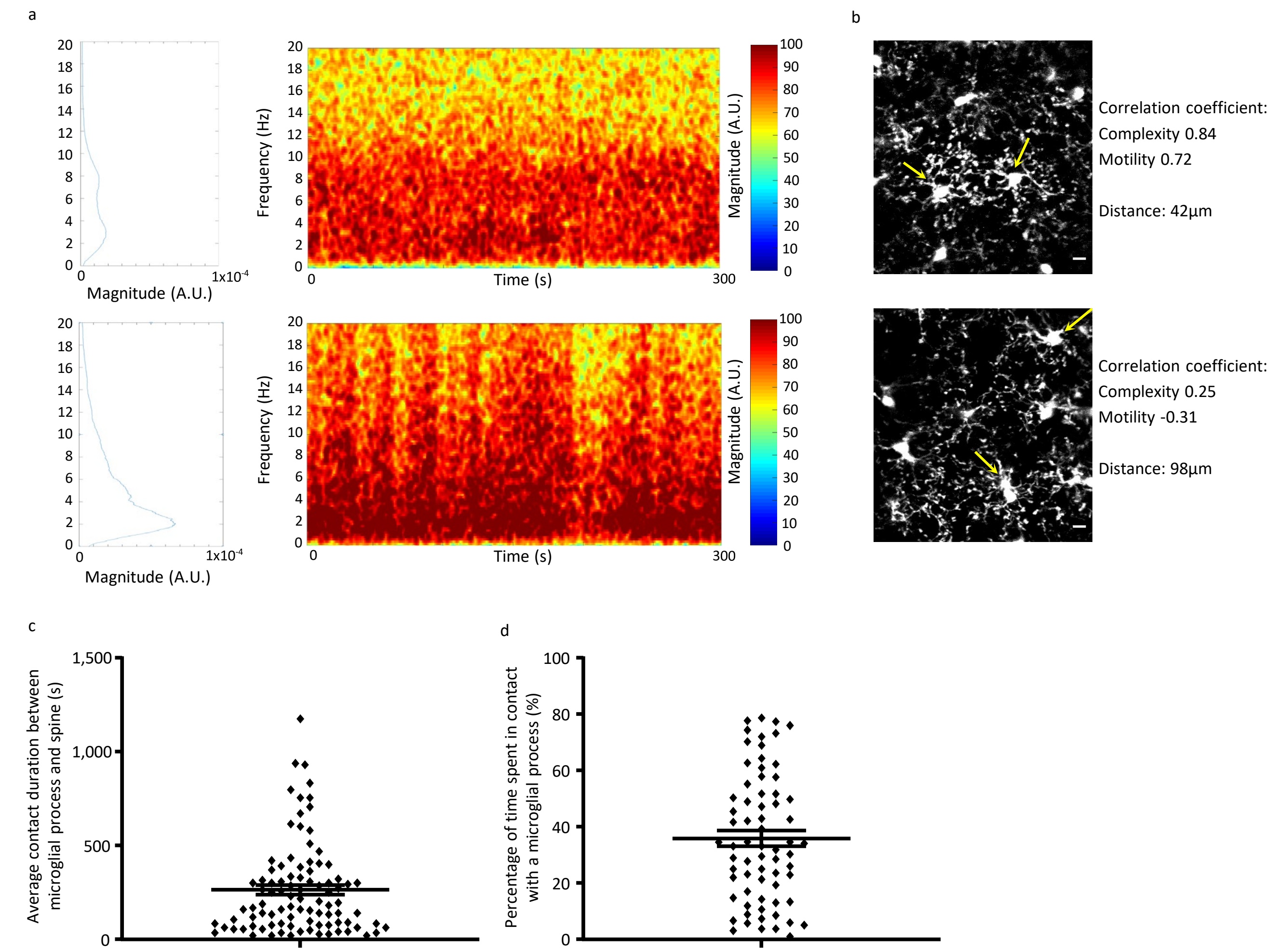

Supplementary Figure 2. Characterization of the impact of global and local neuronal activity on microglial morphodynamics. (a) Characteristic power spectrum (left panel) and color-coded time-frequency graph (right panel) for low-amplitude and high-amplitude EEG signal. **(b)** Examples of two microglial cells that are close (upper panel) or far apart (lower panel) with their corresponding correlation coefficients for complexity and motility. Representative images from the analysis present in Figure 1f-1g, replicated 20 times. **(c)** Average duration of microglia-spine contact (n=48 spines from 5 mice, 7-14 spines/mouse). **(d)** Average percentage of time the spine spends in contact with microglial processes (n=68 spines from 5 mice, 11-22 spines/mouse). All data are represented as mean ± SEM. Source data are provided as a Source Data file for (c-d).

SUPPLEMENTARY FIGURE 3

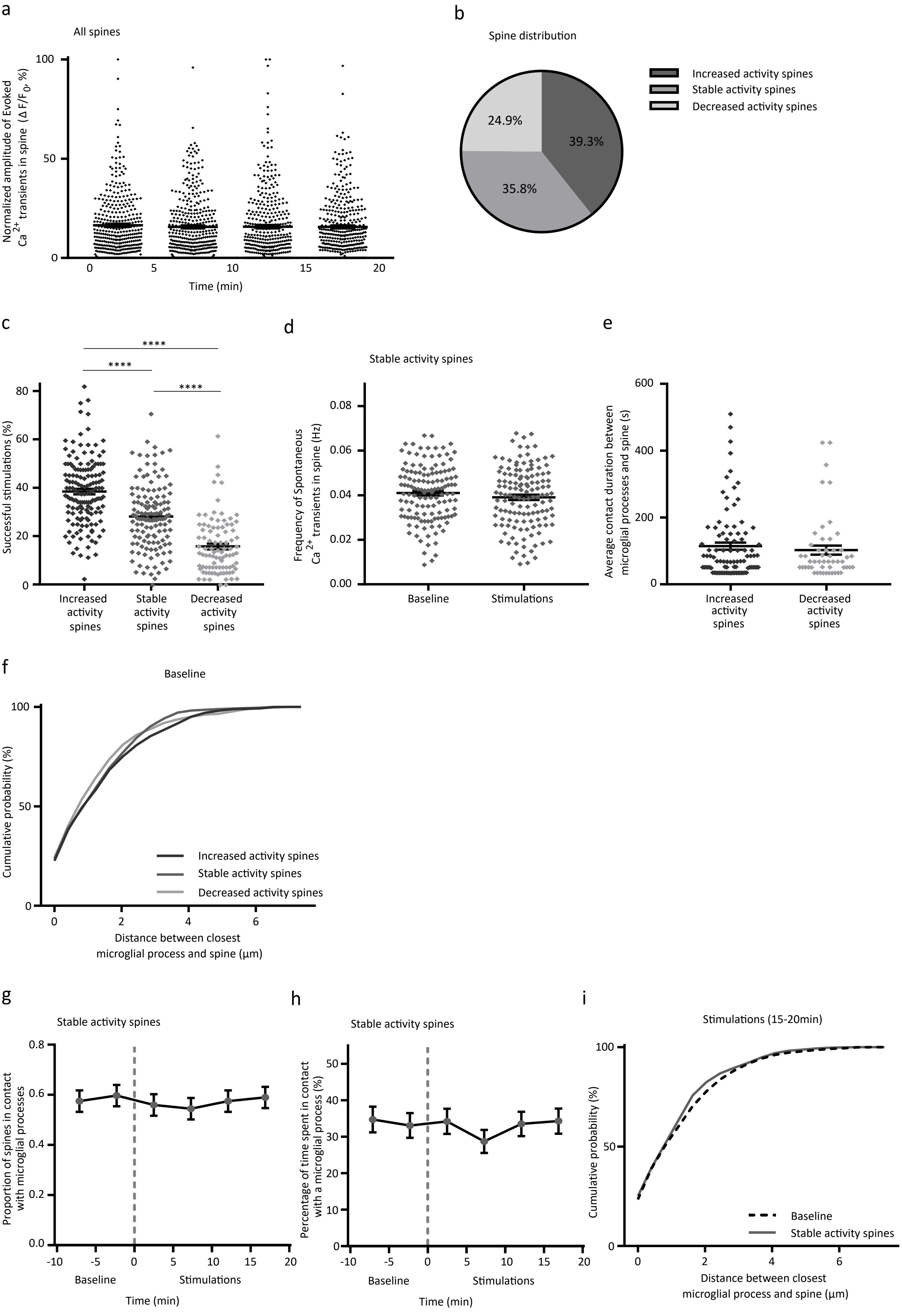

Supplementary Figure 3. Calcium transients characterization in spines during whisker stimulation. (a) Average amplitude of the evoked Ca^{2+} transients in spine over the stimulation period (n=374 spines from 3 mice, one-way ANOVA, p>0.05). **(b)** Spine distribution based on their ratio of Ca^{2+} fluctuations ($\int \Delta F/F_0 dt$) between the 10-minute period during stimulations and baseline: n_{IA}=147 spines (39.3%), n_{SA}=134 spines (35.8%) and n_{DA}=93 spines (24.9%) from 3 mice. **(c)** Average success rate of individual spines in response to the stimulations based on their spontaneous response to the stimulations (n_{IA}=147 spines, n_{SA}=134 spines and n_{DA}=93 spines from 3 mice, two tailed one-way ANOVA, ****p<0.0001). **(d)** Average spontaneous frequency of Ca^{2+} events for individual spines calculated before and during the stimulation period for Stable activity spines (n_{SA}=134 spines from 3 mice, paired t-test, two-tailed, p=0.0521). **(e)** Average duration of microglia-spine contact during the baseline period over a period of 10 minutes (n_{IA}=92 contacts and n_{DA}=51 contacts from 3 mice, unpaired t-test, two-tailed, p=0.5051). **(f)** Cumulative probability of the distance between the closest microglial process for Increased activity spines (dark line), Stable activity spines (mid-light line) and Decreased activity spines (light line) for a 5-minute episode during the baseline period (n_{IA}=147 spines, n_{SA}=134 spines and n_{DA}=93 spines from 3 mice, Kolmogorov-Smirnov test, p>0.05). **(g)** 5-minute episodes showing the kinetics of the proportion of spines contacted by microglial processes for Stable activity spines (n_{SA}=134 spines from 3 mice, one-way ANOVA, p>0.05) and **(h)** average percentage of time individual spines spent in contact with microglial processes for Stable activity spines (n_{SA}=134 spines from 3 mice, one-way ANOVA, p>0.05). **(i)** Cumulative probability of the distance between the closest microglial process for Stable activity spines (mid-light line) for a 5-minute episode during the stimulation period (baseline shown with dashed line) (n_{SA}=134 spines from 3 mice, Kolmogorov-Smirnov test, p>0.05). All data are represented as mean ± SEM. Source data are provided as a Source Data file for (a-i).

Supplementary Figure 4

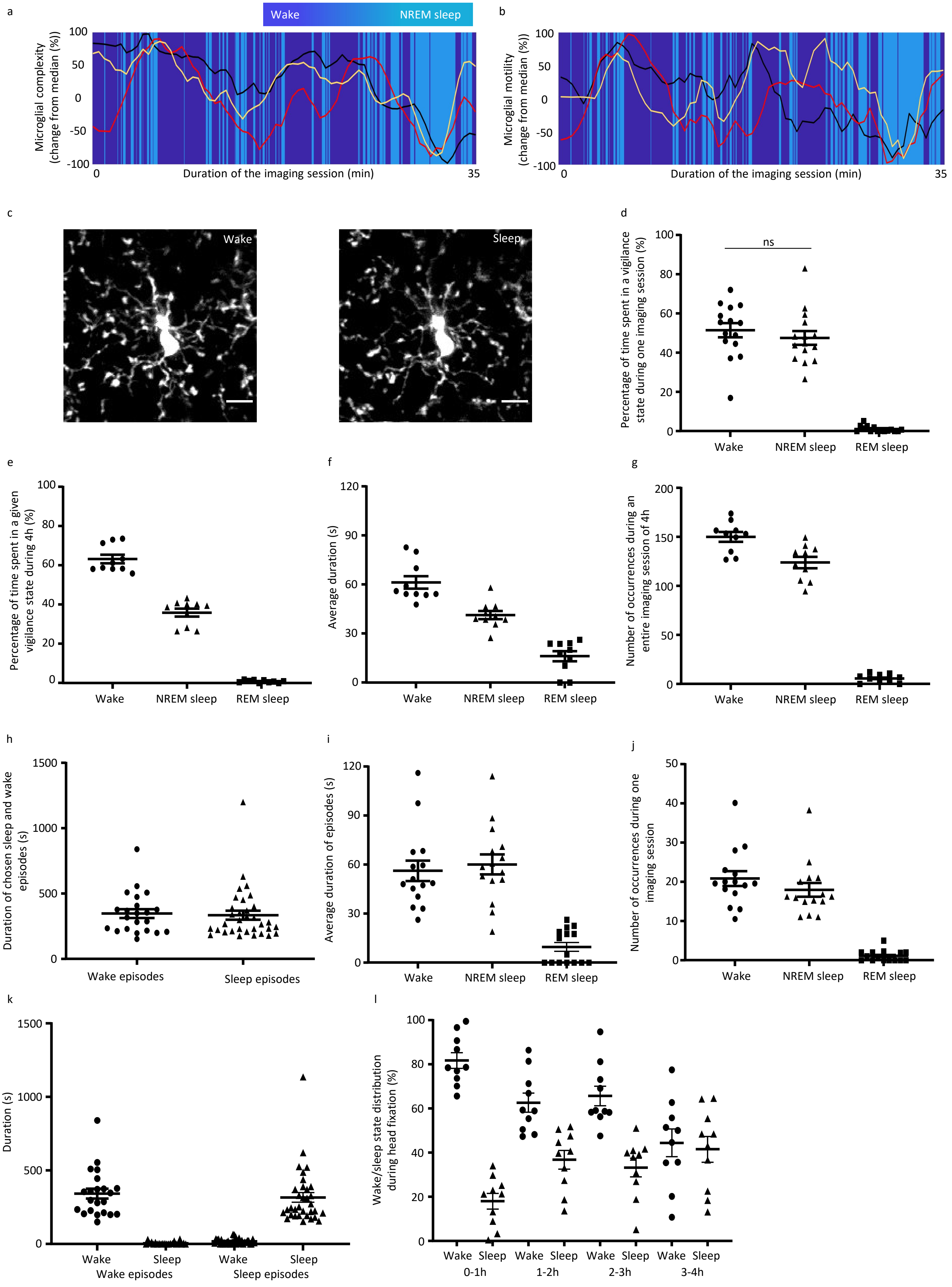

Supplementary Figure 4. Characterization of sleep and wake properties in head-restrained mice, and their impact on microglial morphodynamics. (a, b) Evolution of the **(a)** complexity and **(b)** motility for three microglial cells (yellow, red and black lines) during an entire imaging session containing several episodes of wake and sleep. **(c)** Raw images showing the color-coded microglial cell from Figure 4c during wake and sleep. Representative images from the analysis present in Figure 4d-4e, replicated 30 times. **(d)** Percentage of time spent awake or in NREM and REM sleep during one imaging session of 35 minutes (n=15 sessions from 6 mice, Wilcoxon test for wake and sleep comparison, two-tailed, p>0.05). **(e, f, g)** Quantification of wake and sleep (NREM and REM) from 4-hour recordings in head-restrained mice. **(e)** Percentage of time spent during wake, sleep and REM sleep during a 4-hour session (n=10 4-hour sessions from 6 mice). **(f)** Average duration of individual wake, NREM and REM sleep episodes during a 4-hour session (n=10 4-hour sessions from 6 mice). **(g)** Total number of wake, NREM and REM sleep episodes with varying lengths over a 4-hour session (n=10 4-hour sessions from 6 mice). **(h)** Average duration of wake and sleep episodes chosen for morphology and motility quantification (n=22 episodes of wake and n=33 episodes of sleep from 6 mice). **(i, j)** Quantification of sleep and wake during 35-minute imaging sessions. **(i)** Average duration of individual wake, NREM and REM sleep episodes for one imaging session of 35 minutes (n=15 sessions from 6 mice). **(j)** Total number of wake, NREM and REM sleep episodes over 35-minute imaging sessions (n=15 sessions from 6 mice). **(k)** Average duration of wake and sleep from chosen wake and sleep episodes (n=22 episodes of wake and n=33 episodes of sleep from 6 mice). **(l)** The distribution of the percentage of wake and sleep during the 4h head-fixation sessions (n=10 4-hour sessions from 6 mice). All data are represented as mean ± SEM. Source data are provided as a Source Data file for (d-l).

Supplementary Figure 5

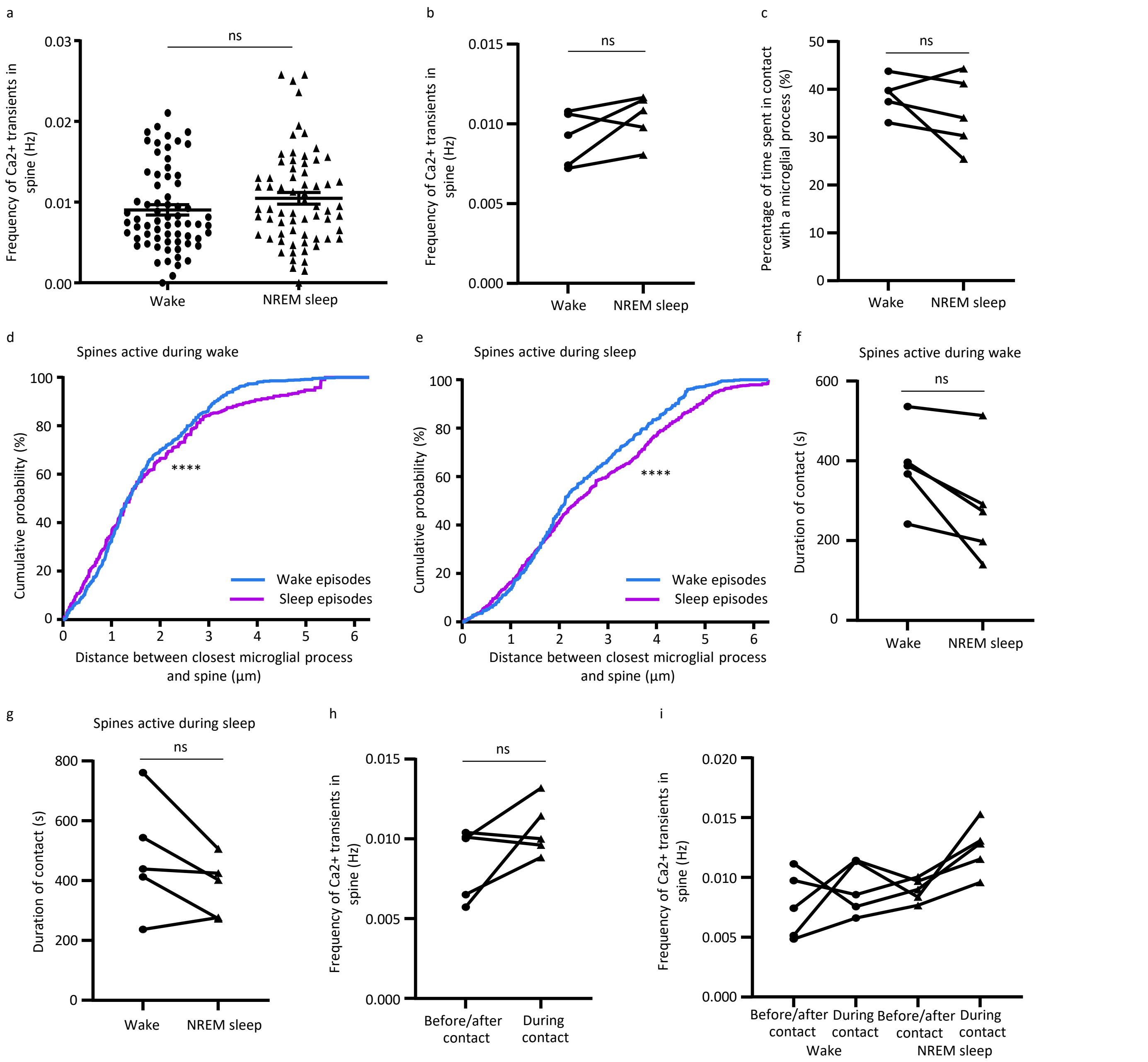

Vake NREM sleep

Supplementary Figure 5. Characterization of the microglia-spine distance relative to the activity of the spine and the vigilance states. (a) Average frequency of spine Ca^{2+} transients during wake and during sleep (n=11-22 spines from 5 mice, paired t-test, two-tailed, p=0.106). **(b)** Average frequency of spine Ca^{2+} transients during wake and during sleep (n=5 mice, Wilcoxon test, two-tailed, p=0.125). **(c)** Percentage of time spent by the spine in contact with a microglial process during wake and sleep (n=5 mice, Wilcoxon test, two-tailed, p=0.438). **(d, e)** The cumulative distribution of microglia-spine distance during **(d)** wake episodes and **(e)** sleep episodes for spines active during wake and spines active during sleep (Kolmogorov-Smirnov test, two-tailed, ****p<0.0001). **(f, g)** For episodes of wake and sleep, duration of contact between microglial processes and **(f)** spines active during wake (n=5 mice, Wilcoxon test, two-tailed, p=0.0625) and **(g)** spines active during sleep (n=5 mice, Wilcoxon test, two-tailed, p=0.188). **(h)** Frequency of spine Ca^{2+} transients during or before/after contact with microglial processes (n=5 mice, Wilcoxon test, two-tailed, p=0.312). **(i)** Frequency of spine Ca^{2+} before/after and during microglial contact during episodes of wake and sleep (n=5 mice, Friedman test; p>0.05, except for comparison between spine Ca^{2+} frequency before/after contact during episodes of wake vs spine Ca^{2+} frequency during contact during episodes of sleep, *p=0.02). All data are represented as mean ± SEM. Source data are provided as a Source Data file for (a-i).
