# Supplementary material for: Sleep decreases neuronal activity control of microglial dynamics in mice
Source: Nat Commun. 2022 Oct 21;13:6273. doi: 10.1038/s41467-022-34035-9 (PMC9586953; doi:10.1038/s41467-022-34035-9)
Supplement: Supplementary file 3 — Reporting Summary [file 41467_2022_34035_MOESM3_ESM.pdf]

nature portfolio

Corresponding author(s): Olivier Pascual
Last updated by author(s): Oct 7, 2022

Reporting Summary

Nature Portfolio wishes to improve the reproducibility of the work that we publish. This form provides structure for consistency and transparency in reporting. For further information on Nature Portfolio policies, see our Editorial Policies and the Editorial Policy Checklist.

Statistics

For all statistical analyses, confirm that the following items are present in the figure legend, table legend, main text, or Methods section.

n/a	Confirmed	
☐	☒	The exact sample size (*n*) for each experimental group/condition, given as a discrete number and unit of measurement
☐	☒	A statement on whether measurements were taken from distinct samples or whether the same sample was measured repeatedly
☐	☒	The statistical test(s) used AND whether they are one- or two-sided<br>*Only common tests should be described solely by name; describe more complex techniques in the Methods section.*
☒	☐	A description of all covariates tested
☒	☐	A description of any assumptions or corrections, such as tests of normality and adjustment for multiple comparisons
☐	☒	A full description of the statistical parameters including central tendency (e.g. means) or other basic estimates (e.g. regression coefficient) AND variation (e.g. standard deviation) or associated estimates of uncertainty (e.g. confidence intervals)
☐	☒	For null hypothesis testing, the test statistic (e.g. *F*, *t*, *r*) with confidence intervals, effect sizes, degrees of freedom and *P* value noted<br>*Give P values as exact values whenever suitable.*
☒	☐	For Bayesian analysis, information on the choice of priors and Markov chain Monte Carlo settings
☒	☐	For hierarchical and complex designs, identification of the appropriate level for tests and full reporting of outcomes
☐	☒	Estimates of effect sizes (e.g. Cohen's *d*, Pearson's *r*), indicating how they were calculated

Our web collection on statistics for biologists contains articles on many of the points above.

Software and code

Policy information about availability of computer code

Data collection	Images and electrophysiological acquisitions were done using Prairie View (version 5.5), while custom-written software (Arduino) was used to trigger whisker -stimulations.
Data analysis	Images processing and analysis were performed using Image J (NIH, version 1.53t), custom-written MatLab (version R2018a) and LabVIEWview software (National Instruments, version 2020.0.1).<br>Statistical analysis waswere performed using GraphPad Prism (version 7.0).

For manuscripts utilizing custom algorithms or software that are central to the research but not yet described in published literature, software must be made available to editors and reviewers. We strongly encourage code deposition in a community repository (e.g. GitHub). See the Nature Portfolio guidelines for submitting code & software for further information.

Data

Policy information about availability of data

All manuscripts must include a data availability statement. This statement should provide the following information, where applicable:

- Accession codes, unique identifiers, or web links for publicly available datasets
- A description of any restrictions on data availability
- For clinical datasets or third party data, please ensure that the statement adheres to our policy

All the data supporting the findings of this study are available in the manuscript. Due to the size of all raw source files, they are not available on a public data repository. However, raw source files and scripts are both available upon request.

Human research participants

Policy information about studies involving human research participants and Sex and Gender in Research.

Reporting on sex and gender	N/A
Population characteristics	N/A
Recruitment	N/A
Ethics oversight	N/A

Note that full information on the approval of the study protocol must also be provided in the manuscript.

Field-specific reporting

Please select the one below that is the best fit for your research. If you are not sure, read the appropriate sections before making your selection.

[x] Life sciences [ ] Behavioural & social sciences [ ] Ecological, evolutionary & environmental sciences

For a reference copy of the document with all sections, see nature.com/documents/nr-reporting-summary-flat.pdf

Life sciences study design

All studies must disclose on these points even when the disclosure is negative.

Sample size	No statistical methods were used prior to experimentation, however sample size was initially defined in accordance with previously published experiments using similar experimental design (Hristovska et al 2020) and based on our laboratory experience of the model.
Data exclusions	Two mice were excluded prior to EEG power profile analysis due to bad EEG implantation that could lead to misinterpretation in the analysis. As stated in the manuscript, outliers, corresponding to value out of the physiological range observed, were identified and removed from the analysis by using Grubbs' test.
Replication	Experiments were replicated multiple times, with the number of replication clearly indicated in the text and figures legend of the figures. All attempts at replication are present in the manuscript and were successful. In addition to this, morphodynamics analysis during sleep and wake episodes were successfully replicated by two independent experimenters.
Randomization	No formal randomization of mice was performed for this study since there was no drug or treatment comparisons within the same age group. The randomization is not relevant in our study because each measurement was compared to the baseline. Comparison were performed within individuals so it is not possible to randomize the mice that are all from the same age, sex and come from the same animal facility.
Blinding	Experiments comparing heterozygous and homozygous mice were not performed blindly because the phenotype was visible to the experimentator and needed some adjustments during the imaging session to be comparable at the end. However, the quantification parameters and data processing methods were strictly defined prior to quantification to avoid any bias and analysis was performed blindly.

Reporting for specific materials, systems and methods

We require information from authors about some types of materials, experimental systems and methods used in many studies. Here, indicate whether each material, system or method listed is relevant to your study. If you are not sure if a list item applies to your research, read the appropriate section before selecting a response.

Materials & experimental systems

n/a	Involved in the study
☒	☐ Antibodies
☒	☐ Eukaryotic cell lines
☒	☐ Palaeontology and archaeology
☐	☒ Animals and other organisms
☒	☐ Clinical data
☒	☐ Dual use research of concern

Methods

n/a	Involved in the study
☒	☐ ChIP-seq
☒	☐ Flow cytometry
☒	☐ MRI-based neuroimaging

Animals and other research organisms

Policy information about studies involving animals; ARRIVE guidelines recommended for reporting animal research, and Sex and Gender in Research

Laboratory animals	All strains of mice were on a C57Bl/6J background and 6 to 10 week-old male mice were used.<br>Cx3cr1-eGFP (JAX: 005582) and Cx3cr1-CreERT2 x ROSA26-STOP-tdTomato (JAX: 021160 and JAX: 007905, both heterozygous for Cx3cr1 expression, were used to visualize microglial cells during in vivo two photon imaging sessions. Cx3cr1-eGFP homozygous mice were used in comparison to heterozygous ones to evaluate the impact of Cx3cr1 invalidation.<br>The animals were housed at animal facilities, in ventilated and enriched cages (bedding and running wheels), at 22+/-2°C, under a light/dark cycle of 12 hours (light onset at 07:00 am) and were given access to food and water ad libitum.
Wild animals	The study did not involve wild animals
Reporting on sex	Only males were used in our experiments because of a more stable hormonal status without sterilization, and because of heavy experiments whichthat restrict to a low number of subjects. We do not exclude different results in females.
Field-collected samples	The study did not use field collected samples.
Ethics oversight	All animal procedures were conducted in accordance with the Guidelines of the Animal Care Facility of University Claude Bernard Lyon 1 and were approved by French Ministry of Agriculture (Apafis #DR2014-14, Apafis #7839, Apafis #10350 and Apafis #20983) and Local Ethics Committee CE2A55.

Note that full information on the approval of the study protocol must also be provided in the manuscript.
